# Spillover of viruses and global concern: is simian haemorrhagic fever virus next in queue? – correspondence

**DOI:** 10.1097/MS9.0000000000000438

**Published:** 2023-04-04

**Authors:** Shailesh K. Patel, Ankush K. Niranjan, Nikhil K. Channabasappa, Jigyasa Rana, Aditya Agrawal, Talha B. Emran

**Affiliations:** a Department of Veterinary Pathology; b Department of Veterinary Microbiology; c Department of Veterinary Physiology and Biochemistry, College of Veterinary Science and Animal Husbandry, Rewa, Madhya Pradesh; d Department of Veterinary Anatomy, Faculty of Veterinary and Animal Sciences, Rajiv Gandhi South Campus, Banaras Hindu University, Barkachha, Mirzapur, Uttar Pradesh, India; e Department of Pharmacy, BGC Trust University Bangladesh, Chittagong; f Department of Pharmacy, Faculty of Allied Health Sciences, Daffodil International University, Dhaka, Bangladesh

Highlights

- Simian Haemorrhagic Fever Virus (SHFV) poses a great threat to African primates by causing fatal haemorrhagic fever.
- Researchers have reported CD163, as an intracellular receptor for SHFV.
- Replication competency of SHFV in human monocytes further increases the concern.

*Dear Editor*,

The global population is witnessing the emergence of infectious zoonotic diseases due to altered environmental homeostasis by humans at the peak of evolution. Remarkably, primitive microbes mutate and adapt according to the need or turmoil. Anthropogenic activities, such as civilisation, industrialisation, and deforestation, have brought humans and wild animals in close proximity, resulting in the spillover of new pathogens. Diseases like Influenza, Ebola, Marburg, Zika, Boca, SARS-CoV (severe acute respiratory syndrome coronavirus), MERS-CoV (Middle East respiratory syndrome coronavirus), Lassa, Nipah, and Hendra, originating in response to natural infringement, have spread globally. Additionally, many pathogens are on the verge of spillover to humans after a long and continuous adaptation process, owing to continuous contact with human cells followed by a series of mutations. Many distinctive pathogens circulate among domestic and wild animals worldwide without causing symptoms. An increasing number of unique pathogens, like animal viruses, have jumped to humans and wreaked havoc on naive immune systems by unravelling, gaining access to cells, multiplying, and escaping the body’s defence mechanisms. The Simian Haemorrhagic Fever Virus (SHFV), endemic in monkeys and some African primates, poses a great risk of spillover in the near future.

SHFV is a single-stranded, positive-sense RNA virus within the family *Arteriviridae*, first isolated in 1964 from rhesus macaques. Arteriviruses include equine arteritis virus, porcine reproductive and respiratory syndrome virus, SHFV, lactate dehydrogenase-elevating virus, and wobbly possum disease virus, and their hosts include horses, pigs, possums, nonhuman primates, and rodents. Various African nonhuman primates, such as patas monkeys, vervets, Guinea baboons, and olive baboons, have been implicated as potential carriers^1^,^2^. SHFV infection is generally asymptomatic in nonhuman primates, but is an acute, highly fatal infection in Asian macaques, characterised by disseminated vascular damage, intravascular coagulation, and severe haemorrhagic lesions throughout the body. SHFV infection is highly contagious in macaques and animal-to-animal transmission is mainly reported via aerosols, infected fomites, or direct contact. While SHFV is already considered a critical threat to nonhuman primates, human infections have not been reported yet. Hence, the impact of species-jumping is still uncertain.

SHFV poses a great threat to African primates by causing fatal haemorrhagic fevers and jumping species barriers. Interestingly, researchers have reported CD163 as an intracellular receptor for SHFV, which serves as a rare mode of virus entry shared with other haemorrhagic fever viruses such as Ebola virus and Lassa virus^3^. Additionally, the tropism and replication competency of SHFV in human monocytes signify the presence of operational cellular proteins for replication. Furthermore, after host switching, at least three distinct simian arteriviruses have already caused deaths in macaques. Thus, SHFVs in nature may not require major adaptations to the human host.

Remarkably, profound similarities have been reported in SHFVs and simian viruses responsible for the human immunodeficiency virus (HIV) pandemic. Like HIV and simian immunodeficiency virus (SIV), SHFV has also been reported to successfully evade human cells by disabling protective immune mechanisms, thus taking long-term hold in the body (https://www.news-medical.net/news/20220930/Obscure-family-of-viruses-already-endemic-in-African-monkeys-poised-for-spillover-to-humans.aspx). These findings suggest that it is necessary to monitor SHFV to avoid another possible pandemic.

Although SHFV infections have not been reported in humans, studies on simian arteriviruses must be prioritised by global health communities, considering its spillover potential. High arterivirus viral loads have been reported in asymptomatic African monkeys, which frequently interact with human populations. HIV has been reported to originate from SIVs in African nonhuman primates and jumped to humans during the early 1900s. It caused fatalities in humans in the 1980s in the United States, but no serological tests or treatments were available. Thus, although jumping the species barrier in SHFV is uncertain, more animal viruses are likely to spread to humans. As humans are immunologically naive to SHFV, the development of serological tests for serosurveillance of high-risk populations in close contact with animal carriers is needed. Moreover, raising awareness and continuously monitoring viruses with zoonotic potential is critical for the rapid containment of possible future spillovers.

Globalisation, human settlements in forests, and increased international travel have enabled the rapid spread of pathogens than ever before. Historically, it took years or even decades for diseases to spread between countries and continents, allowing many previously unexplored regions of the world to remain disease-free; for example the Black Death happened over 150 years before Columbus’s first expedition to the Americas, leaving these continents largely unaffected. Based on the ‘One Health’ perspective and experiences from past epidemics and pandemics, drivers of spillover events can be impeded by strategic planning^4^. Thus, constant and methodical applications are required to prevent spillover incidents, as these are the only ways to prevent future epidemics.

## Ethical approval

Not applicable.

## Consent

Not applicable.

## Sources of funding

None.

## Author contribution

All the authors substantially contributed to this work.

## Conflicts of interest

There are no conflicts of interest.

## Provenance and peer review

Not commissioned, externally peer-reviewed.
